# Modelling the health impact and cost-effectiveness of lymphatic filariasis eradication under varying levels of mass drug administration scale-up and geographic coverage

**DOI:** 10.1136/bmjgh-2015-000021

**Published:** 2016-04-06

**Authors:** Christopher M Stone, Randee Kastner, Peter Steinmann, Nakul Chitnis, Marcel Tanner, Fabrizio Tediosi

**Affiliations:** 1 Swiss Tropical and Public Health Institute, Basel, Switzerland; 2 Universität Basel, Basel, Switzerland; 3 Department of Statistics, North Carolina State University, Raleigh, North Carolina, USA.

## Abstract

**Background:**

A global programme to eliminate lymphatic filariasis (GPELF) is underway, yet two key programmatic features are currently still lacking: (1) the extension of efforts to all lymphatic filariasis (LF) endemic countries, and (2) the expansion of geographic coverage of mass drug administration (MDA) within countries. For varying levels of scale-up of MDA, we assessed the health benefits and the incremental cost-effectiveness ratios (ICERs) associated with LF eradication, projected the potential savings due to decreased morbidity management needs, and estimated potential household productivity gains as a result of reduced LF-related morbidity.

**Methods:**

We extended an LF transmission model to track hydrocele and lymphoedema incidence in order to obtain estimates of the disability adjusted life years (DALYs) averted due to scaling up MDA over a period of 50 years. We then estimated the ICERs and the cost-effectiveness acceptability curves associated with different rates of MDA scale-up. Health systems savings were estimated by considering the averted morbidity, treatment-seeking behaviour and morbidity management costs. Gains in worker productivity were estimated by multiplying estimated working days lost as a result of morbidity with country-specific per-worker agricultural wages.

**Results:**

Our projections indicate that a massive scaling-up of MDA could lead to 4.38 million incremental DALYs averted over a 50-year time horizon compared to a scenario which mirrors current efforts against LF. In comparison to maintaining the current rate of progress against LF, massive scaling-up of MDA—pursuing LF eradication as soon as possible—was most likely to be cost-effective above a willingness to pay threshold of US$71.5/DALY averted. Intensified MDA scale-up was also associated with lower ICERs. Furthermore, this could result in health systems savings up to US$483 million. Extending coverage to all endemic areas could generate additional economic benefits through gains in worker productivity between US$3.4 and US$14.4 billion.

**Conclusions:**

In addition to ethical and political motivations for scaling-up MDA rapidly, this analysis provides economic support for increasing the intensity of MDA programmes.

**Key questions**

**What is already known about this topic?**

- A key challenge for the elimination of lymphatic filariasis (LF) is the expansion of geographic coverage of mass drug administration programmes. Without intense scale-up, elimination of LF will require both more time and more treatments.
- Prior studies have not considered the cost-effectiveness associated with scaling-up geographic coverage of an eradication programme, while accounting for progress made to date in eliminating LF.

**What are the new findings?**

- The faster geographic coverage of mass drug administration programmes is brought to scale, the greater the health benefits will be in terms of disability adjusted life years averted (DALY).
- Extending coverage to all endemic countries, including those that to date have not yet undertaken mass drug administration programmes, was most likely to be cost-effective above a willingness to pay threshold of US$71.5/DALY averted when scale-up occurred at the fastest rate.

**Recommendations for policy and practice**

- The Global Programme to Eliminate Lymphatic Filariasis has been succesful to date, but it has been recognised that coverage will have to be scaled up substantially if the target of elimination by 2020 is to be achieved. This analysis suggests that more intense forms of scale-up are most likely to be cost-effective, lending further support to intensifying LF elimination efforts.

## Introduction

To date, smallpox is the only human infectious disease that has been eradicated through deliberate efforts, an accomplishment that is considered among the greatest medical achievements in the past century.^1^ This success increased interest in disease eradication as a public health strategy, and eradication campaigns against poliomyelitis and dracunculiasis (Guinea worm) are currently underway. Progress against dracunculiasis indicates that the concept of eradication can be applied to parasitic infections for which vaccines are not available.^2^

Disease eradication results from the permanent cessation of transmission of the causative agent of the disease globally and the ultimate disappearance of the organism as a free-living biological species. This is distinct from elimination, which is the interruption of transmission in a defined geographic area, mostly a country or a region. Control reflects the use of interventions aimed at reducing the health burden associated with transmission of a pathogen, but does not intend to interrupt transmission.^3^ The decision to shift from a strategy based on reducing the health burden to one of elimination and progressively aiming at eradication is not to be taken lightly.^4^ Since eradication is an all-or-nothing achievement, and one that will require an intensified and/or altogether different strategy than disease control, failure to achieve it may represent a misuse of resources. In addition, failed attempts lead to donor fatigue with persistent negative consequences.^5^ To provide policymakers with guidance on whether to pursue eradication, the concept of an Eradication Investment Case (EIC) was developed following insights from an Ernst Strüngmann forum on scientific advances in disease eradication.^6^ ^7^ An EIC is expected to include a quantitative assessment of the technical and biological feasibility of achieving eradication, an assessment as to whether the health system infrastructure is capable of delivering the interventions, and evidence of sufficient funding and political will to support such a programme. The various components also need to be periodically re-evaluated as the programme progresses, since all are potentially prone to erosion due to factors including emerging drug resistance, weak health systems, or public and donor fatigue.^8^

Further arguments for or against engaging in eradication will come from economic considerations.^9^ Using a game-theoretic approach to the eradication of smallpox, Barrett and Hoel^10^ were able to specify conditions under which an eradication strategy was optimal. Specifically, pursuing an intensive control strategy was never optimal, if eradication was possible. Similar arguments based on health economic modelling have been made to support continued investments in the eradication of poliomyelitis.^11^

Lymphatic filariasis (LF) endemicity is strongly tied to poverty^12^ and leads to debilitating, chronic forms of morbidity, most notably hydrocele and lymphoedema.^13^ The health burden from LF is considerable, estimated at 2.77 million disability adjusted life years (DALYs) (1.8–4.0 million) in 2010.^14^ Beyond affecting physical health and productivity, LF-related morbidity also leads to stigma and social exclusion, and impacts mental well-being.^15^ Such exclusion can lead to delays in treatment seeking, diminish prospects for marriage or employment or interfere with the ability of school-aged children to attend school.^16–18^ This, in turn, may exacerbate the link to poverty for families with one or more infected household members.

Preventive chemotherapy represents the primary strategy of the ongoing Global Programme to Eliminate Lymphatic Filariasis (GPELF), which aims for eradication of LF by 2020.^19^ The strategy is based on once-yearly mass drug administration (MDA) either with diethylcarbamazine citrate (DEC) and albendazole (ABZ), or, in areas where onchocerciasis is also endemic, ivermectin (IVM) and ABZ. These compounds kill microfilariae and affect the survivorship and/or fecundity of adult worms.^20^ If MDA is provided to a large proportion of the population (>65%) for a sufficient number of years, interruption of transmission in the targeted region is thought to be feasible.^13^

As LF proceeds towards elimination and eradication, certain challenges are worthy of consideration, including the feasibility of reaching remote populations and the ability to maintain coverage in urban areas with dense and mobile populations.^21^ An animal reservoir is not generally thought to contribute to LF transmission, although *Brugia malayi* is sometimes found in non-human primates, cats and dogs.^22^ In Central African areas coendemic with *Loa loa*, it remains to be seen if the provisional strategy based on vector control and twice-yearly MDA with ABZ will be successful.^19^ For the purposes of this study we assume that eradication of LF is feasible and the mentioned challenges can be tackled.

We previously developed scenarios that could lead to the elimination or eradication of LF, estimated the time it might take to reach elimination and eradication, projected the number of treatments required under each scenario, and considered the associated financial and economic costs.^23^ In the current study, we assess the health impact in terms of disability-adjusted life years (DALYs) averted, estimate the cost-effectiveness associated with different intensities of scaling-up MDA, and project the possible savings to the health system and potential increase in worker productivity due to averted LF-related morbidity for each of these scenarios.

## Methods

### Scenarios modelled

We defined four scenarios which differ in their geographic coverage and rate of MDA scale-up. The scenarios were developed in an iterative consensus process involving leading scientists, policymakers, programme managers and other stakeholders following an analysis of the on-going GPELF.^23^ For areas co-endemic with *Wuchereria bancrofti *and* Loa loa* we made the simplifying assumption that whatever strategy will end up being used in reality (eg, the provisional guidelines from the WHO suggest bi-annual MDA of ABZ and vector control) can be approximated in our model by annual MDA with IVM+ALB. The current elimination scenario is defined as the comparator scenario, mirroring the rate of MDA scale-up seen under the GPELF thus far, but assuming that countries that have not yet begun MDA programmes will not do so. As we identified low levels of geographic coverage within certain endemic areas to be the major impediment to progress against LF, the three eradication scenarios explore the impact of expanding MDA to all LF endemic populations at varying rates. Eradication I models the impact of expanding MDA to all endemic areas at the historical average rate of scale-up; eradication II assumes countries scale-up geographic coverage by 20% increments each year, and eradication III represents the best-case scenario, whereby all countries expand coverage to their entire at-risk population immediately. See the SI and Kastner *et al*^23^ for further details.

### Estimates of DALYs

We extended a previously published deterministic model, EpiFil,^24^ which simulates filariasis transmission by either *Anopheles* species or *Culex* species vectors.^25^ See the SI for details on how the model was expanded to include chronic disease states.

We translated the incidence of chronic disease to DALYs, which, in the case of LF, are composed of the years of life lived with a disability (YLD) multiplied by the disability weight (DW). We determined the number of new hydrocele and lymphoedema cases in a given time period and assigned YLDs at that point based on the individual's remaining life expectancy.^26^ Per convention, no distinction in the DW was made between lymphoedema and hydrocele, and symptomatic cases were assigned a DW of 0.11.^27^ Age-weighting was not considered in this study, but DALYs were discounted at 3% per year. Further details on the calculations are provided in the SI. The DALYs were estimated for a period of 50 years to capture the long-term health benefits of interrupting transmission.

### Estimates of financial and economic costs

Programmatic costs were estimated from the perspective of an LF-endemic country's health system, with future costs discounted at 3%. Activities considered in the cost estimates included advocacy, capacity strengthening, coordination and strengthening partnerships, data management, on-going surveillance, monitoring, evaluation and supervision, drug delivery, and administration. Costs for mapping, running post-MDA transmission assessment surveys and increased surveillance in areas of *L. loa* prevalence were also taken into account. A summary of the costing methodology is provided in the online supplementary material.

10.1136/bmjgh-2015-000021.supp1 Supplementary data

### Cost-effectiveness analysis

In order to evaluate the cost-effectiveness of eradication, the DALY projections for each country in each scenario were paired with country-specific cost estimates. With the elimination scenario as the reference case against which all other scenarios could be compared, cost-effectiveness was assessed using incremental cost-effectiveness ratios (ICERs). For each simulation, the monetary net benefits (MNB) were calculated as the mean incremental DALYs averted multiplied by the decision makers’ maximum willingness to pay for a DALY averted minus the mean incremental cost for the scenario.^28^ Cost-effectiveness acceptability curves were used to graphically depict the probability for each scenario to be cost-effective at various willingness-to-pay thresholds.

### Potential health system savings and worker productivity losses

To assess the potential health systems savings due to averted morbidity management, we followed the approach of Chu *et al* and assumed that on average 40% (20–50%) of hydrocele patients and 50% (30–55%) of patients with lymphoedema seek treatment annually. We further assumed acute adenolymphangitis (ADL) to occur about twice per year (0–7 times) in 70% (45–90%) of hydrocele patients, and four (0–7 times) times annually for 95% (90–95%) of patients with lymphoedema.^29^ Since it remains uncertain to what extent treatment with DEC and ABZ or IVM and ABZ reduces clinical manifestations, we assume treatment seeking remains at the same level during the course that patients remain symptomatic. The estimated savings are therefore possibly conservative.

Health systems savings were then estimated by combining the averted incidence of morbidity, frequency of ADL episodes and treatment-seeking behaviour paired with country-specific costs for a consultation at a primary health centre with 50% population coverage, taken from the most recent update of the WHO CHOICE database.^30^ Parameter uncertainty was considered by taking 500 random estimates within each parameter range, assuming normal distributions for treatment-seeking behaviour and triangular distributions for ADL episodes.

Using a pre-established methodology, we also determined the impact that LF eradication could have on worker productivity.^29^ To assess the potential worker productivity increase, we assumed ADL episodes to last 4 days on average (1–9 days), and cause a 75% (50–93%) reduction in productivity for their duration. LF-related morbidity was assumed to decrease the amount of productive working days by 15% (13–17%) for hydrocele patients and 20% (15–22%) for those with lymphoedema. We monetarily valued possible gains in worker productivity by taking the number of working days lost due to LF-related morbidity paired with country-specific (when available) or region-specific daily per-worker agriculture wages given by the World Bank's World Development Indicators Online, inflated to 2012. Other databases may provide different estimates to value the productivity of people with LF, many of whom are subsistence farmers.^29^ Uncertainty in the productivity-related parameter estimates was therefore incorporated by drawing 500 random samples from each range assuming normal distributions. All results were discounted at 3%.

### Role of the funding source

The study sponsor had no role in study design; in the collection, analysis and interpretation of data; in the writing of the report; and in the decision to submit the paper for publication.

## Results

### Estimates of DALYs

**Figure 1:**
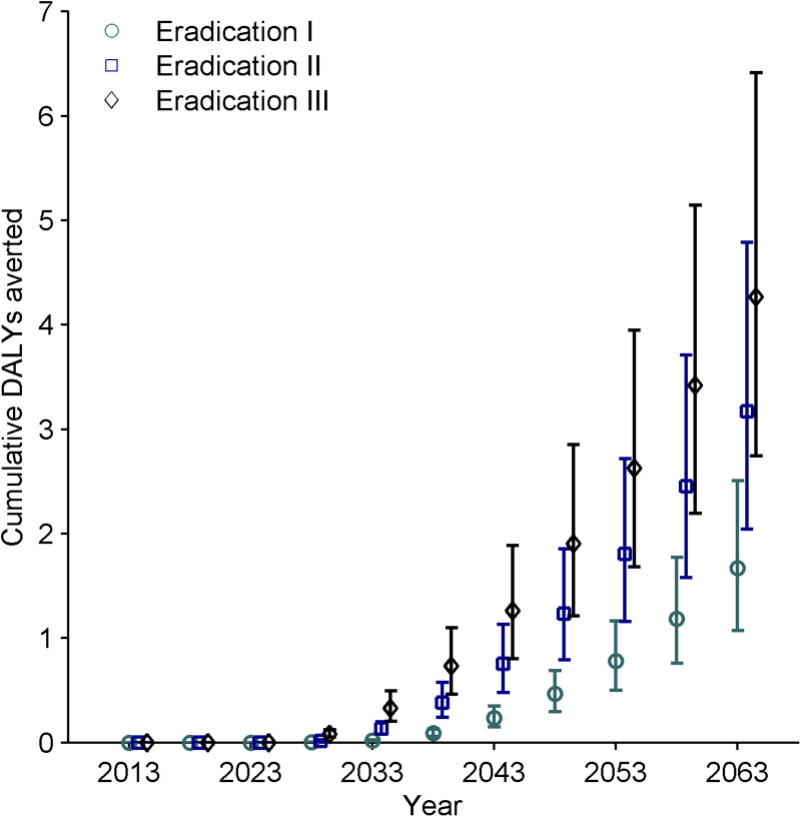
Cumulative number of DALYs averted over time per eradication scenario compared to the elimination scenario. DALY, disability adjusted life years.

The intensity of MDA scale-up greatly impacts population health (figure 1). With elimination as the comparator, extending MDA to all endemic countries (eradication I) results in approximately 1.72 million DALYs averted (95% Credible Interval (CrI) 1.09–2.61 million) over a 50-year time horizon. In contrast, intensifying geographic coverage in all countries (eradication III) leads to approximately 4.38 million DALYs averted (95% CrI 2.78–6.5 million) over the same timeframe. Thus, there are considerable gains to achieve by more intensely scaling-up MDA (table 1).

**Table 1:** Summary of key results with 95% Credible Intervals

	Elimination	Eradication I	Eradication II	Eradication III
Number of treatments (millions)	3.41 (3.18–3.53)	4.66 (4.41–4.90)	4.37 (4.13–4.59)	4.16 (3.92–4.38)
DALYs averted (millions)*	–	1.72 (1.09–2.62)	2.98 (1.90–4.45)	4.38 (2.79–6.50)
Financial costs (millions USD)	929.2 (883.5–971.5)	1289.4 (1226.7–1344.9)	1273.5 (1208.9–1331.4)	1234.9 (1172.3–1299.8)
Economic costs (billions USD)	5.19 (4.91–5.45)	7.91 (7.50–8.300)	7.97 (7.55–8.37)	7.53 (7.12–7.94)
ICER (USD/DALY averted)*	–	219.0 (142.65–322.72)	120.7 (79.47–177.70)	72.94 (47.74–109.80)
Potential savings to health system (millions USD)*	–	139.9 (63.8–260.3)	335.6 (152.2–626.8)	483.4 (219.1–902.6)
Potential gains in worker productivity (billions USD)*	–	3.41 (2.03–5.36)	10.06 (5.98–15.50)	14.43 (8.58–22.02)

*Measured against the elimination scenario as the comparator.

DALY, disability adjusted life years; ICER, incremental cost-effectiveness ratios.

**Figure 2:**
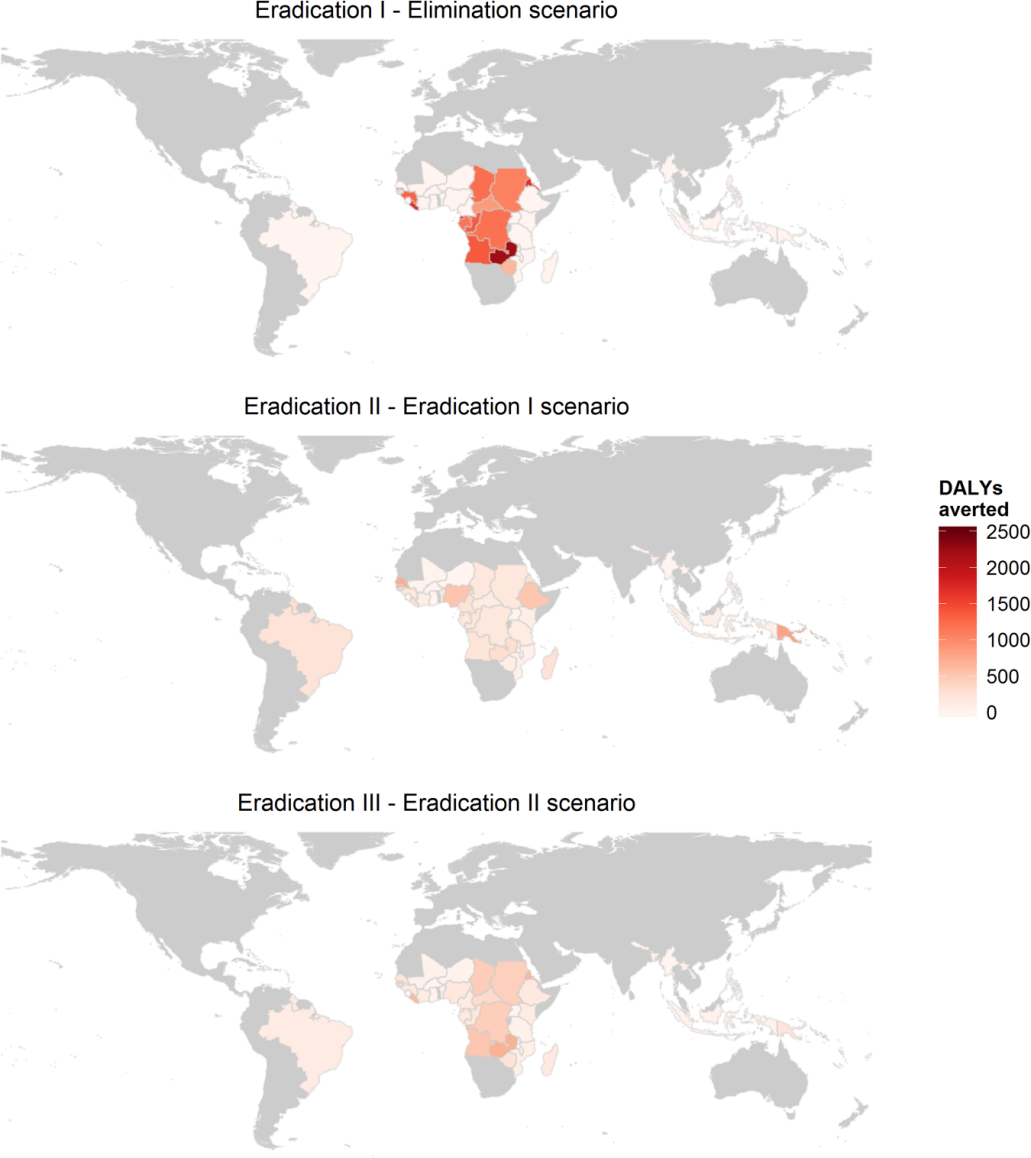
Cumulative number of DALYs averted per 100 000 persons after 50 years per country, comparing the different scenarios to each other. DALY, disability adjusted life years.

The incremental health impacts by country, expressed as DALYs averted per 100 000 people, are depicted for the eradication I scenario compared to the elimination scenario, eradication II compared to eradication I and eradication III compared to eradication II (figure 2). The comparison between eradication I and the elimination scenario illustrates that the majority of the gains from extending MDA to all endemic countries are concentrated in Central Africa. The heterogeneous results within these countries are largely due to demographic patterns that affect the DALY estimates, such as age composition, life expectancy and population growth rates. The gains from increasing the rate of MDA scale-up are more evenly spread out among countries (eradication II vs I, and III vs II).

### Cost-effectiveness analysis

**Figure 3:**
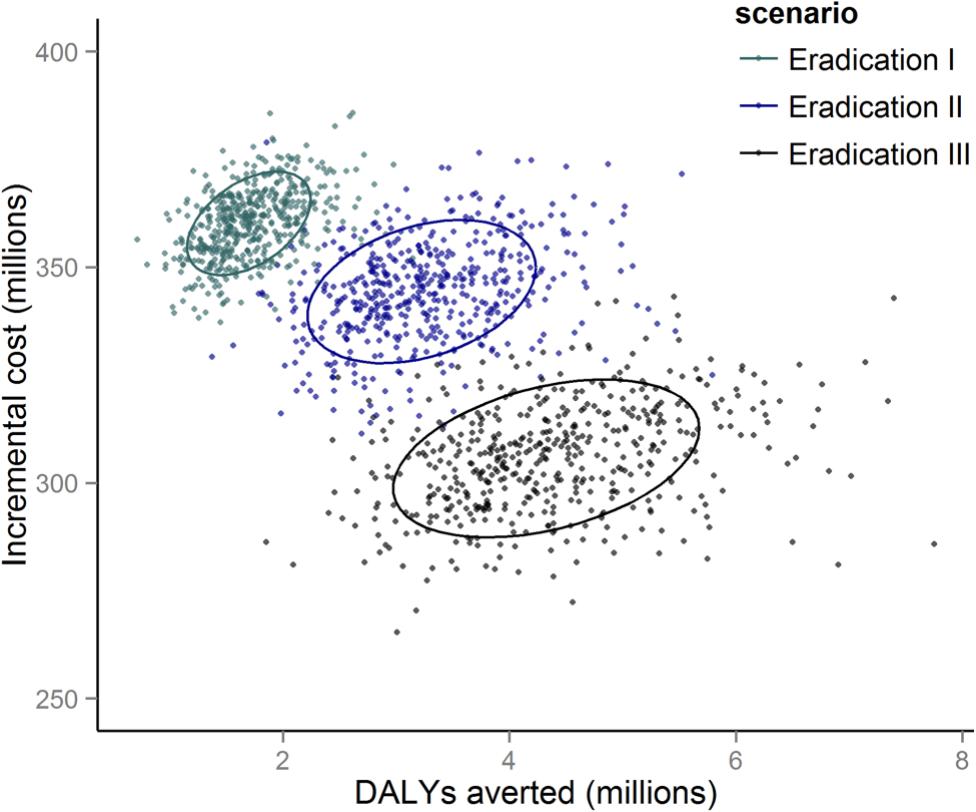
Incremental cost-effectiveness plane and 95% CI ellipses with incremental financial costs associated with MDA programmes and incremental disability adjusted life years averted, comparing the three eradication scenarios to the comparator scenario. DALY, disability adjusted life years; MDA, mass drug administration.

**Figure 4:**
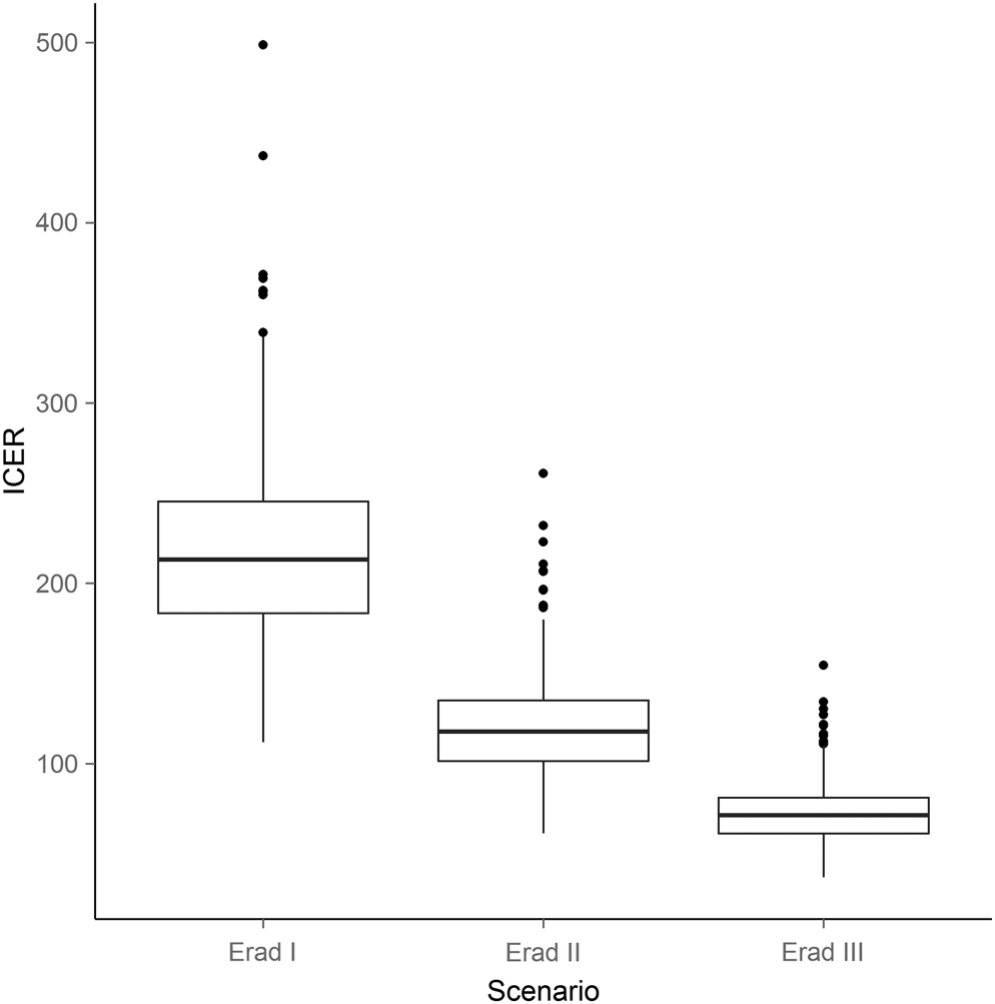
Incremental cost-effectiveness ratios associated with each of the scenarios, with elimination as the comparator. DALY, disability adjusted life years.

**Figure 5:**
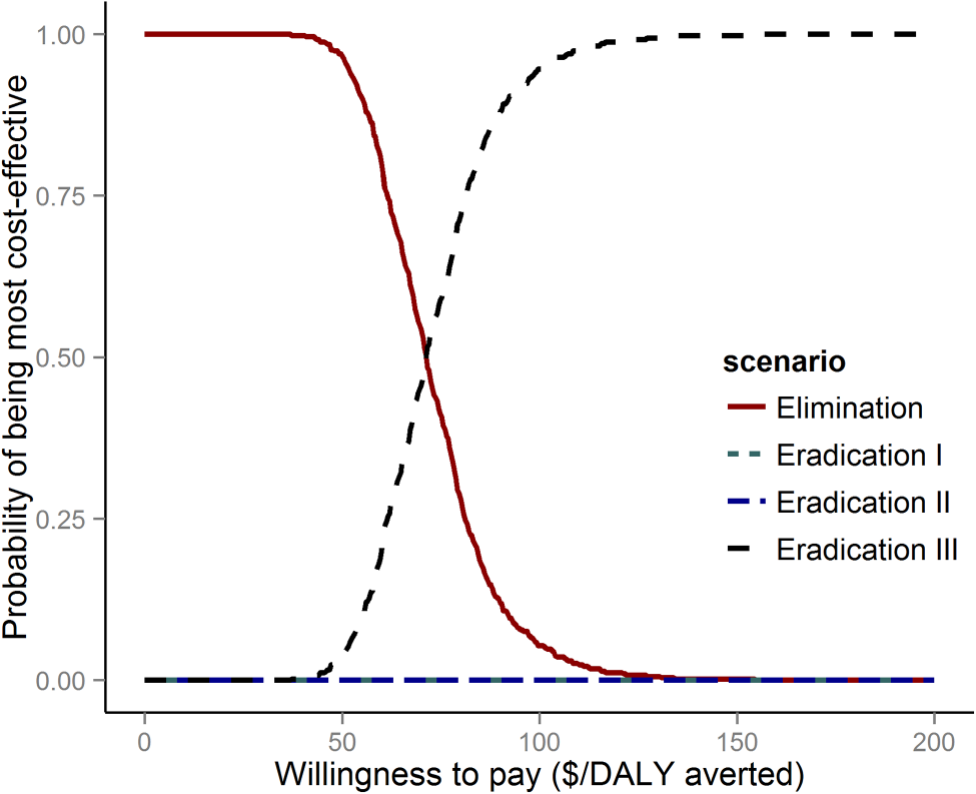
Cost-effectiveness acceptability curve for the four scenarios highlighting the uncertainty around cost-effectiveness ratios. Above the cost-effectiveness threshold of $71.50/DALY the probability of the eradication III scenario being more cost-effective than the elimination scenario increases. When eradication III is a realistic option, eradication scenarios I and II are never the most cost-effective. DALY, disability adjusted life years.

The estimated ICER for the eradication III scenario is approximately US$73/DALY averted (95% CrI US$47.7–US$109.8/DALY averted; figures 3 and 4). In contrast, the eradication I and eradication II scenarios are higher, at US$219/DALY averted (95% CrI US$142.7–US$322.7/DALY averted) and US$121/DALY averted (95% CrI US$79.5–US$177.7/DALY averted), respectively. Against the elimination scenario, all eradication scenarios end in the northeast quadrant of the incremental cost-effectiveness plane (figure 3), which implies an increase in DALYs averted at increased cost.^31^ Correspondingly, and as shown by the cost-effectiveness acceptability curve, if the willingness to pay threshold would surpass US$71.5/DALY averted, scale-up of MDA to all at-risk populations in all endemic countries should be pursued as quickly as possible (figure 5).

### Potential health system savings and worker productivity losses

**Figure 6:**
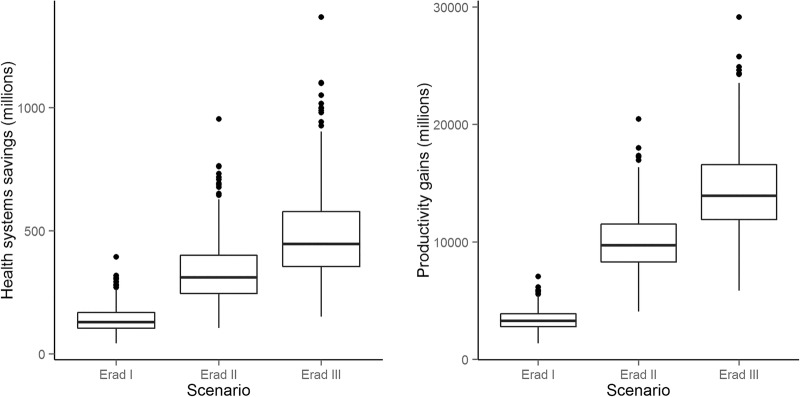
Cumulative cost savings and averted losses over 50 years associated with LF eradication scenarios. Left: potential cost savings to LF endemic health systems due to decreased need for morbidity management practices; right: averted productivity losses due to eradication. LF, lymphatic filariasis.

Unsurprisingly, reaching LF eradication sooner was found to correspond to increased health systems savings, due to decreased morbidity management, ranging from US$140 million (95% CrI US$53.8–US$260.3m) in the eradication I scenario to US$483 million (95% CrI US$219.1–US$902.6m) in eradication III (figure 6).

Potential savings to the health system, however, were dwarfed by possible gains in worker productivity, which ranged from approximately US$3.4 billion (95% CrI US$2.03–US$5.36bn) under the eradication I scenario to US$14.4 billion (95% CrI US$8.58–US$22.02 billion) in the eradication III scenario (figure 6). Importantly, all increased with increasing rates of MDA scale-up, further supporting the conclusion from the cost-effectiveness analysis.

## Discussion

LF could become the first vector-borne disease to be eradicated. While the GPELF has made notable progress thus far, in order to achieve eradication, the programme needs to be extended to several endemic countries. Moreover, if the goal of global elimination as a public health problem by 2020, as specified in the London Declaration,^32^ is to occur, the scale-up of MDA to cover all populations at risk needs to be greatly intensified.

Here, we estimate that the impact on the health burden due to LF will increase with the rate of MDA scale-up, since DALYs averted have a longer time period to accrue when transmission is interrupted earlier. This highlights the importance of measuring costs and benefits of interventions over a long time horizon, as well as the benefits of integrating disease transmission, economic and demographic models.

Intensifying the rate of MDA scale-up to eradicate LF is clearly supported on economic grounds. Our analysis suggests that above a willingness to pay threshold of US$71.5/DALY averted, pursuing eradication at the highest level of MDA scale-up is the most likely to provide the greatest net benefits and therefore provide the most value for money. To put this in perspective, a willingness to pay of US$150/DALY averted has been suggested for low and middle income countries as acceptable.^33^ While decision makers are not bound by this threshold, our analysis indicates that LF eradication would generally be considered cost-effective, assuming the rate of MDA scale-up is sufficient. If instantaneous scale-up (eradication III) is shown not to be feasible, the ICER of the eradication II scenario (rapid scale-up) remains low at US$121/DALY averted. Only at the slowest level of scale-up does the ICER fall above this threshold, adding further urgency to intensifying the rate of scale-up. Others have used the Gross National income per capita for low income counties of US$1035 as a threshold,^34^ by which measure all eradication scenarios are considered cost-effective. Cost-effectiveness as a measure of efficiency is typically applied to interventions or health programmes. Additionally, it has been suggested that less efficient programmes may be considered in the case of eradication (as opposed to disease control) programmes, due to a host of additional outcomes that are typically not captured in cost-effectiveness analyses.^35^ These could include the threats of resistance, insecurity of long-term funding, or implications for economic growth.^36^

Other considerations influence the cost-effectiveness of LF eradication. Depending on the perspective taken, the benefits that are expected to arise due to health systems savings and gains in worker productivity could be taken into account, which would further increase the dominance of the eradication III scenario. We did not consider certain aspects of morbidity management, such as the need for hydrocele surgeries, which would diminish over time as transmission is interrupted. The economic benefits of eradication could therefore be greater than estimated here. Likewise, our estimates of gains in productivity are likely conservative, because they were based on the time lost due to LF-related morbidity and agricultural wages, rather than on direct estimates of output and productivity loss (which are reported to be greater for LF, though data is scarce).^29^ By tracking morbidity only for hydrocele and lymphoedema, but not subclinical outcomes such as lymphatic dilation, or clinical manifestations such as ADL or tropical pulmonary eosinophilia, or a potential for excess mortality (either due to a lack of data or a lack of disability weights), we underestimate the true burden of disease.^37^ There are some epidemiological aspects that we did not consider, such as recrudescence of infections in areas following elimination due to migration. By ignoring this possibility, we made the implicit assumption that international movement among endemic populations was limited. Relaxing this assumption would require a metapopulation model and an investigation of human migration and commuting patterns in LF-endemic regions. However, previous studies in which similar mechanisms were considered have only added to the growing support for pursuing eradication.^10^ ^11^

Further aspects which could interfere with the ability to maintain sufficiently high MDA coverage include insufficient political will, inadequate health infrastructure, logistical issues and the potential of systematic non-compliance. The development of drug resistance, as has been documented in animal systems,^38^ could also present complications. Further and equally important, in areas where *W. bancrofti* is co-endemic with *L. loa*, it remains to be seen how effective biannual distribution of ABZ by itself or together with long-lasting insecticidal nets will be. We have assumed that the strategy employed in these areas would be as effective as MDA with IVM and ABZ, and as unlikely to lead to resistance. However, if this is not the case, and an alternative strategy requires a larger investment or a prolonged campaign, the ICERs of the eradication scenarios will increase. We have likewise not accounted for any progress in interrupting LF transmission resulting from bed net programmes targeting malaria, although modelling suggests such methods are highly efficacious against LF.^39^ ^40^ If vector control is going to be part of a strategy against LF in certain regions, cost estimates should likewise incorporate this intervention. Further, it is worth noting that our estimates of progress made to the current time were informed by the WHO PCT Databank, as described in Kastner *et al*.^23^ It has been pointed out that these self-reported values are sometimes overestimates of the true coverage.^41^ Accounting for this bias would likely decrease the ICERs of the eradication scenarios. Currently, data to improve on these estimates is unavailable but additional modelling work, more focused on individual districts based on local data, may be enlightening. Such work will be particularly valuable in identifying more effective strategies for dealing with endemic districts where progress seems to be lagging. Such strategies could potentially include novel technologies, or novel combinations, such as a proposed triple-drug treatment regime.^42^

Additionally, we assumed that endemic countries implemented MDA programmes for a fixed duration resulting in a high probability of achieving elimination (ie, where >97.5% of simulations reached elimination).^23^ A more dynamic decision process, whereby a shorter duration is followed by surveys and possible additional rounds of MDA until elimination is certified may be closer to reality, but beyond the scope of this global-level exercise.

Finally, our strategies assumed that all endemic countries included in the different scenarios are committed to elimination, and would not pursue a less ambitious goal, such as disease control only. It is plausible, however, for some countries to only target populations that live in moderate to high transmission zones, but not the greater number of people in low transmission areas where chronic disease is much less prevalent. A previous study indeed suggests that cost-effectiveness may improve if communities with microfilaria prevalence above 3.55% are first treated through a sequential strategy based first on control and a later shift of programme goals towards elimination.^43^ Ordering the treatment districts by intensity could thus lead to further increases in cost-effectiveness of our eradication scenarios.

In conclusion, this study suggests that eradication of LF is likely a cost-effective strategy and that if pursued, scaling up MDA as rapidly as feasible will result in increases in value.
