# Supplementary material for: Modelling the health impact and cost-effectiveness of lymphatic filariasis eradication under varying levels of mass drug administration scale-up and geographic coverage
Source: BMJ Glob Health. 2016 Apr 6;1(1):e000021. doi: 10.1136/bmjgh-2015-000021 (PMC5321305; doi:10.1136/bmjgh-2015-000021)
Supplement: Supplementary data [file bmjgh-2015-000021supp.pdf]

Supplementary material

Scenarios

The lymphatic filariasis (LF) elimination and eradication scenarios are based on an analysis of the currently ongoing elimination programme. The composition of the scenarios have previously been described in detail.[1] Briefly, the scenarios were based on the mass drug administration (MDA) of filaricidal drugs: albendazole (ABZ) either in combination with diethylcarbamazine citrate (DEC) or, in areas where coendemicity of *Onchocerca volvulus* is a concern, with ivermectin (IVM).

To account for heterogeneity in transmission intensity within countries, we assigned proportions of each at-risk population to transmission archetypes of approximately 0% (i.e., the proportion of implementation units (IUs) that were false positive), 5%, 10%, 15% and 20% microfilaremia prevalence. The data reflect the situation in African countries, i.e., the proportion of endemic implementation units that fall within >0-5% prevalence, >5-10%, >10-15%, or >15%. The archetypes used in the analysis reflect an upper boundary to the underlying data, save for the highest level, where a 20% prevalence was taken as a representation of very high transmission levels. The average proportion of IUs that fell within these categories was extrapolated to all other countries for which these data were not available. Uncertainty in this estimate was included by treating these averages as a probability in a multinomial distribution.[1]

We used the estimated number of treatments required for each year, in each LF-endemic country and each scenario from Kastner *et al*.[1] These estimates were based upon the number of annual treatment rounds of MDA necessary, taking into consideration the underlying microfilaria prevalence quartiles in each country, whether transmission is due to *Anopheles* spp. or *Culex* spp., the drug regimen used, and the number of effective MDA rounds that had already occurred prior to 2013. These estimates are conservative in the sense that they allowed 97.5% of our simulations to achieve elimination. We thus assumed that public health officials implemented MDA programmes of these durations in order to assure a high probability of achieving elimination. We then used these estimated durations to predict the number of treatments required per year for each of the elimination and eradication scenarios, as

well as to develop estimates of the financial and economic costs associated with implementing the scenarios (Kastner *et al.*, submitted).

Transmission model

We used two versions of the deterministic model, EpiFil [2], to simulate adult filarial worm, microfilaria and infective stage larvae (L3) transmission and dynamics when the mosquito vectors were either *Anopheles* or *Culex* spp. The model versions we used largely followed the structure presented by Gambhir & Michael,[3] which includes the possibility of female worms remaining unmated in humans at low densities, and provides different microfilariae uptake functions (facilitation and limitation) for *Anopheles* spp. and *Culex* spp. vectors. We assumed that transmission by other mosquito genera (e.g., *Aedes*, *Mansonia*) is approximated well enough by these model versions.

The transmission model was thus the same as used and described in our related analysis.[1] The model consists of the following partial differential equations used to describe changes in state parameters:

$$\frac{\partial W}{\partial t}+\frac{\partial W}{\partial a}=\lambda\frac{V}{H}\psi_1\psi_2 s_2 h(a)L^* e^{-\beta I}-\mu W \quad (1)$$

$$\frac{\partial M}{\partial t}+\frac{\partial M}{\partial a}=\alpha\phi(W,k)W-\gamma M \quad (2)$$

$$\frac{\partial I}{\partial t}+\frac{\partial I}{\partial a}=W-\delta I \quad (3)$$

The state parameters represent the mean adult worm burden in humans, *W*; the mean microfilariae density in humans, *M*; and the mean level of immunity to infection, *I*. The initial conditions were $W(0,t) = M(0,t) = I(0,t) = 0$, while $W(a,0)$, $M(a,0)$ and $I(a,0)$ were the equilibrium levels in the absence of interventions, obtained numerically by simulating the model for a sufficiently long period. L^*, the mean L3 density in the mosquito population, is given by:

$$L^* = \frac{\lambda\kappa g\int_a \pi(a)(1-f(M))da}{\sigma+\lambda\psi_1} \quad (4)$$

The functions *h(a)* and *π(a)* represent the age-dependent availability to mosquitoes (represented as a linear increase from 0 to 1 over the first 10 years of life and a value of 1 for further years) and an

approximation of the human age distribution ($\pi(a)$ = 0·035 $e^{-0·026\ a}$), respectively, as in Norman et al. [2]. The population-averaged uptake of infective stage filarial larvae by mosquitoes, $f(M)$, is defined as:

$$f(M)_{\mathrm{C}} = (1+\frac{M}{k(M)}(1-e^{-r/\kappa}))^{-k(M)} \tag{5}$$

$$f(M)_{\mathrm{A}} = \left(\frac{2}{(1+\frac{M}{k(M)}(1-e^{-r/k}))^{\mathrm{k(M)}}} - \frac{1}{(1+\frac{M}{k(M)}(1-e^{-2r/k}))^{\mathrm{k(M)}}} \right) \tag{6}$$

with $f(M)_{\mathrm{C}}$ describing the function used for *Culex* spp. [2] and $f(M)_{\mathrm{A}}$ for *Anopheles* spp. [3]. The worm mating function [3, 4] is given by:

$$\phi(W,k) = 1-(1+\frac{W}{2k})^{-\mathrm{k}-1} \tag{7}$$

Disease model

To assess the resulting health impact of the different scenarios, the progression of disease, adapted from the ordinary differential equations of Chan *et al.*,[5] was added to the model of LF transmission. These equations suggest that progression to hydrocele and lymphoedema result directly from damage to the lymphatic system. This damage is assumed to accrue over time as a direct consequence of harbouring adult worms. In our model (equation 8) the prevalence of lymphatic damage thus increases over time and with age as a function of the mean adult worm burden in the community. In the absence of adult worms, we assume that the prevalence of lymphatic damage may slowly diminish, as will the prevalence of hydroceles, but not lymphoedema. Different grades of lymphoedema or hydrocele, or other aspects of disability related to LF, are not accounted for:

$$\frac{\partial D}{\partial t}+\frac{\partial D}{\partial a} = \eta(W(1-\mathrm{D}))-\mathrm{r_d D} \tag{8}$$

$$\frac{\partial L}{\partial t}+\frac{\partial L}{\partial a} = \pi D(1-L) \tag{9}$$

$$\frac{\partial H}{\partial t} + \frac{\partial H}{\partial a} = hD(1 - H) - r_h H \qquad (10)$$

where *D* represents the mean proportion of humans with lymphatic damage; the mean proportion of humans with lymphoedema is given by *L*; and the mean proportion of males in areas where *W. bancrofti* is the causative agent with hydrocele by *H*. The rate parameters and range of prior values used are described in Supplementary Table 1.

Parameter estimation

We used the same posterior parameter estimates for the parameters of the transmission model (i.e, those appearing in equations 1-7) that resulted in stable prevalence levels associated with the transmission archetypes (ca. 5%, 10%, 15%, and 20% prevalence), as well as the expected durations of MDA programmes in the different transmission settings.[1] The effects of filaricidal treatment were included through a once-yearly instantaneous killing of a proportion of adult worms, μ_w, and microfilariae, μ_{mf}, within the proportion of humans (85% of the total population) covered by the programs. Additionally, the fecundity of adult worms, α, was reduced to zero for six to nine months following a round of MDA within treated humans.[6] The exact values of these killing parameters were randomly sampled from their ranges to allow for a degree of uncertainty in the efficacy of MDA programmes.

The parameters related to the disease model (those in equations 8-10) were estimated for each of the four levels of prevalence associated with our archetypes using the same importance-resampling method as previously described, and similar to that which has been used to fit EpiFil to transmission patterns in specific geographic settings.[1, 7, 8] For the purpose of obtaining disease parameter estimates, we fixed the transmission related parameters at the medians of their previously estimated values, while for the disease-related parameters we drew 10,000 random samples from their uninformative prior ranges. A relation between the prevalence of chronic disease states due to LF (hydrocele and lymphoedema) and microfilaria prevalence in given localities has been investigated and these data are presented by Michael et al.[9] and replotted (Fig S1a). The median and interquartile ranges of the data points per our transmission archetypes are depicted in Fig. S1b.

We ran 10,000 simulations and calculated the goodness of fit of each simulation to the data on chronic disease prevalence. Because we required estimates of the prevalence of hydrocele and lymphoedema, rather than a combined prevalence of chronic disease, the total prevalence was decomposed to male and female prevalence following the global estimate of Michael et al. [10], so that $Pr_{cd,male}$ = $Pr_{cd,total}$*1·75 and $Pr_{cd,female}$ = $Pr_{cd,total}$*0·25, where $Pr_{cd,male}$ is equal to Pr_{l+h} and $Pr_{cd,female}$ is equal to Pr_{l}. We therefore make the simplifying assumptions that males and females acquire lymphoedema at equivalent rates, and that co-occurrence of lymphoedema and hydrocele in males is rare. The goodness of fit to a point prevalence level was assessed through a binomial likelihood:

$$L_i = \prod_{s=m,f} \binom{N}{x_s} p_s^{x_s} (1-p_s)^{N-x_s}$$

, where N is the human population size (assumed to be 1,000), p_s the target prevalence levels of disease associated with the transmission archetype, and x_s is the simulated number of afflicted humans for the simulation run with parameter set, i. Male and female populations are indicated by s. We then randomly sampled, with replacement, 500 parameter sets from the original 10,000 sets proportional to their likelihood, $\Delta L_i = \frac{L_i}{\sum L}$ to obtain an approximation of their posterior distribution.[7] The prevalence of chronic LF-induced disease outcomes from sets of 500 simulations using the resampled parameter sets are depicted in Fig. S1c.

Supplementary Table 1: Parameter descriptions and values used

Parameter	*Description*	*Range (priors)*
λ	human biting rate	6-15
v/h	vectors per human	50-500
ψ_1	proportion of L3s leaving per bite	0·3-0·7
$\psi_2 s_2$	proportion of L3s entering puncture & establishing	0·00002-0·004
g	proportion of bites on infected humans leading to infection	0·25-0·5
β	measure of acquired immunity	0·001-0·2
d	decay of immunity	0-0·005

μ	adult worm death rate	0·014-0·007
$\boldsymbol{\alpha}$	adult worm fecundity	0·2-2
δ	mosquito death rate	0·9-6
γ	death rate of mf	0·083-0·125
C	MDA coverage	85%
μ_{mf}	microfilaricidal effect	0·9-0·95 (DEC + ABZ); 0·95-0·99 (IVM + ABZ)
μ_W	macrofilaricidal effect	0·5-0·6 (DEC + ABZ); 0·3-0·4 (IVM + ABZ)
$\mu_{\boldsymbol{\alpha}}$	suppression of worm fecundity	0·9-0·95 (DEC + ABZ); 0·95-0·99 (IVM + ABZ)
κ	saturation constant in L3 uptake function	4-6
r	constant in L3 uptake function	0·04-0·06
k(M)	aggregation parameter	(0·0006-0·002) + (0·01-0·03) M
η	modifies the rate of accrual of lymphatic damage	0·0006-0·1
π	rate of lymphoedema development	2e-6-0·001
h	rate of hydrocele development	2e-5-0·003
r_h	rate of hydrocele autoresolution	0-0·001
r_d	resolution of lymphatic damage	0-0·001

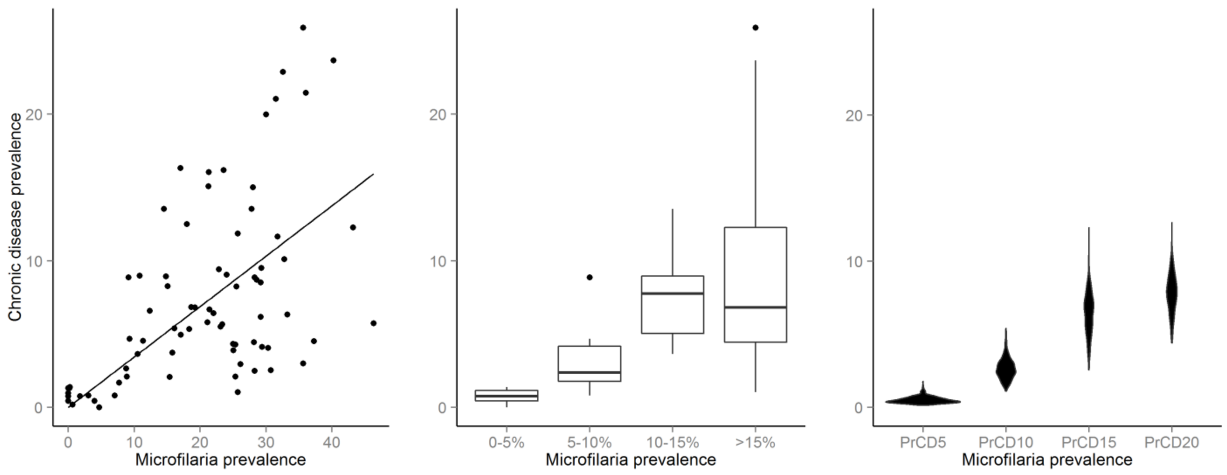

Fig. S1: The relation between prevalence of microfilaremia and chronic disease used to fit disease model parameters. Data points and leftmost panel reproduced from Michael *et al* (2008) .[9] Middle: The median and interquartile ranges of the data points binned per prevalence levels associated with transmission archetypes. Right: Probability densities of chronic disease outcomes from sets of 500 simulations using resampled parameter sets.

Assessing the disability adjusted life years averted by the eradication scenarios

Based on the results of the transmission and disease model, and accounting for the different transmission archetypes and the number of MDA rounds that countries had already completed, the mean prevalence of lymphoedema and hydrocele for each age class over a period of 50 years was computed and recorded as matrices $P_l(t,a)$ and $P_h(t,a)$. In areas where Brugian filariasis predominates, we assumed that males are not affected by hydrocele[11] and (due to paucity of data to improve on this assumption) that lymphoedema progressed as in Bancroftian filariasis. This was implemented for 20% of the Philippines and 60% of Indonesia, based on the ratio of prevalences between types,[11] rounded up or down.

To translate prevalence to incidence per age class, we used country-specific demographic parameters (proportion alive at age x; life expectancy at age x; sex ratio at birth; population growth rate).[12] This resulted in matrices $A_f(t,a)$ and $A_m(t,a)$ which gave the population sizes per five year age class over time, by sex. For each country we calculated the incidence per age per year for both males and females:

$$I_{f,i,j,k}(t,a) = P_{l,i,j,k}(t,a) \circ A_{f,i,j,k}(t,a) \quad (1)$$

$$I_{m,i,j,k}(t,a) = P_{l,i,j,k}(t,a) \circ A_{m,i,j,k}(t,a) + P_{h,i,j,k}(t,a) \circ A_{m,i,j,k}(t,a) \quad (2)$$

where the matrices are multiplied using the Hadamard product (i.e., element-wise), subscript *i* indicates the deciles within a country which differ only in their history of MDA, *j* indicates the four transmission intensity levels, and *k* each of the 500 iterations of the model. We then summed the cases over the deciles, $I_{f,j,k}(t,a)$ and $I_{m,j,k}(t,a)$, and obtained the number of new cases per 5-y-period and 5-y-age-group, as:

$$N_{j,k}(t+l+1,a) = I_{j,k}(t+l+1,a+1) - I_{j,k}(t+l,a) \quad (3)$$

where l = [1, 2,...,10] indicates the 5-y-periods considered. For each age-group and each period, we calculated the years of life lived with disability (YLDs) by multiplying the number of new cases by the remaining expectation of life of that age group, e_l, and the disability weight (DW) of LF-related chronic disease, for males and females (*s*):

$$D_{j,k,s}(t,a) = N_{j,k,s}(t,a)\, e_l dw \frac{1}{dr^t} \quad (4)$$

where the future discounting rate, *dr*, was set to 1·03.

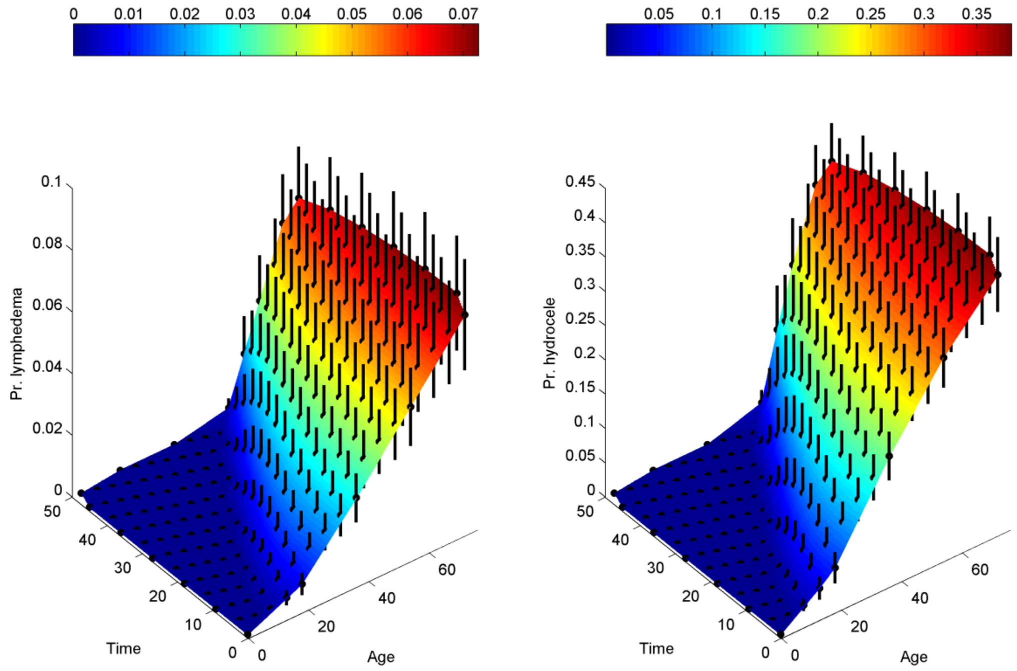

Fig. S2: Mean and standard deviation of 500 simulations of prevalence of lymphoedema and hydrocele for the anopheline model version for 20% microfilaremia over time by age, using once-yearly diethylcarbamazine and albendazole administration for 7 years to interrupt transmission, starting at year 0.

Assessing costs of implementing the global elimination and eradication scenarios

The costs of the scenarios modelled were estimated from the perspective of each LF-endemic country's health system. USAID's *NTD Master Plan Costing Tool in the African Region* from eight AFRO countries were reviewed to assess essential activities and resources needed to undertake the GPELF activities at a country level. Essential activities, resources, and their associated costs were then confirmed by the LF elimination team in Uganda. All costs are reported in 2012 U.S. dollars and discounted at 3%.

Supplementary Table 2: Average worker wage by WHO region

Region	Average daily per worker wage estimate
AFRO	3.61 USD
AMR	25.73 USD
EMR	11.45 USD
SEAR	3.18 USD
WPR	21.02 USD

Using Ugandan costs as a reference, the prospective costs for non-tradable goods and services in all other LF-endemic countries were imputed by adjusting with country-specific purchasing power parity (PPP) conversion factors.[13] All laboratory supplies and capital items were valued at their recommended retails prices. Salaries, as well as prices that were unable to be determined elsewhere, were taken from the WHO-CHOICE databank.[14] Average worker wages and average costs per visit to a health centre per region are listed in supplementary tables 2 and 3. Additional details on the costing methodology can be found in Kastner *et al.* (submitted).

Supplementary Table 3: Average cost per visit to the health system

Region	Average daily per worker wage estimate
AFRO D	6.39 USD
AFR E	7.40 USD
AMR B	19.00 USD
AMR D	11.88 USD
EMR D	8.67 USD
SEAR B	12.44 USD
SEAR D	7.04 USD
WPR A	38.68 USD
WPR B	10.14 USD

References

1. Kastner R, Stone CM, Steinmann P, Tanner M, Tediosi F. What Is Needed to Eradicate Lymphatic Filariasis? A Model-Based Assessment on the Impact of Scaling Up Mass Drug Administration Programs. PloS Neglected Tropical Diseases. 2015; **9**(10): e0004147.
2. Norman R, Chan M, Srividya A, Pani S, Ramaiah K, Vanamail P, et al. The development of an age-structured model for describing the transmission dynamics and control of lymphatic filariasis. Epidemiology and Infection. 2000; **124**: 529-41.
3. Gambhir M, Michael E. Complex ecological dynamics and eradicibility of the vector borne macroparasitic disease, lymphatic filariasis. PLoS ONE. 2008; **3**: e2874.
4. May R. Togetherness among schistosomes: its effects on the dynamics of infection. Mathematical Biosciences. 1977; **35**: 301-43.
5. Chan M, Srividya A, Norman R, Pani S, Ramaiah K, Vanamail P, et al. EPIFIL: a dynamic model of infection and disease in lymphatic filariasis. American Journal of Tropical Medicine and Hygiene. 1998; **59**: 606-14.
6. Michael E, Malecela-Lazaro MN, Simonsen PE, Pedersen EM, Barker G, Kumar A, et al. Mathematical modelling and the control of lymphatic filariasis. The Lancet Infectious Diseases. 2004; **4**(4): 223-34.
7. Gambhir M, Bockarie M, Tisch D, Kazura J, Remais J, Spear R, et al. Geographic and ecologic heterogeneity in elimination thresholds for the major vector-borne helminthic disease, lymphatic filariasis. BMC Biology. 2010; **8**: 22.
8. Singh B, Bockarie M, Gambhir M, Siba P, Tisch D, Kazura JW, et al. Sequential modelling of the effects of mass drug treatments on anopheline-mediated lymphatic filariasis infection in Papua New Guinea. PLoS ONE. 2013; **8**: e67004.
9. Michael E, Malecela M, Zervos M, Kazura JW. Global eradication of lymphatic filariasis: the value of chronic disease control in parasite elimination programmes. PLoS ONE. 2008; **3**: e2936.
10. Michael E, Bundy A, Grenfell B. Re-assessing the global prevalence and distribution of lymphatic filariasis. Parasitology. 1996; **112**: 409-28.
11. Michael E, Bundy D, Grenfell B. Re-assessing the global prevalence and distribution of lymphatic filariasis. Parasitology. 1996; **112**: 409-28.
12. WHO. Life tables for WHO member states. Geneva; 2012.
13. World Bank Indicators TWB. Price level ratio of PPP conversion factor (GDP) to market exchange rate. 2014: (last accessed: 01-3-2015).
14. WHO. Choosing interventions that are cost effective (WHO-CHOICE). Geneva.
